# Keeping an eye on the conductor: neural correlates of visuo-motor synchronization and musical experience

**DOI:** 10.3389/fnhum.2015.00154

**Published:** 2015-04-02

**Authors:** Kentaro Ono, Akinori Nakamura, Burkhard Maess

**Affiliations:** ^1^Human Brain Research Center, Kyoto UniversityKyoto, Japan; ^2^Max Planck Institute for Human Cognitive and Brain SciencesLeipzig, Germany; ^3^National Center for Geriatrics and GerontologyAichi, Japan

**Keywords:** fMRI, sensorimotor synchronization, tapping, musical experience, mental simulation

## Abstract

For orchestra musicians, synchronized playing under a conductor’s direction is necessary to achieve optimal performance. Previous studies using simple auditory/visual stimuli have reported cortico-subcortical networks underlying synchronization and that training improves the accuracy of synchronization. However, it is unclear whether people who played regularly under a conductor and non-musicians activate the same networks when synchronizing with a conductor’s gestures. We conducted a functional magnetic resonance imaging (fMRI) experiment testing nonmusicians and musicians who regularly play music under a conductor. Participants were required to tap the rhythm they perceived from silent movies displaying either conductor’s gestures or a swinging metronome. Musicians performed tapping under a conductor with more precision than nonmusicians. Results from fMRI measurement showed greater activity in the anterior part of the left superior frontal gyrus (SFG) in musicians with more frequent practice under a conductor. Conversely, tapping with the metronome did not show any difference between musicians and nonmusicians, indicating that the expertize effect in tapping under the conductor does not result in a general increase in tapping performance for musicians. These results suggest that orchestra musicians have developed an advanced ability to predict conductor’s next action from the gestures.

## Introduction

When listening to a symphony in a concert hall, we enjoy the music and admire the ability of the musicians to stay in excellent synchrony. How do orchestra musicians achieve such a high level of synchrony? Here we focus on the role of the conductor in a large orchestra. To produce a satisfactory performance, musicians follow the temporal cues provided by the conductors’ gestures. Do orchestra musicians develop a special ability to read the conductors intentions or are they simply good at synchronized action in general? First of all in this introduction we discuss current findings in simple tapping tasks with mechanical pace makers. Second, we briefly review the field of joint action and interpersonal synchrony, and the brain regions that are activated during a tapping task. Finally, the choice of our experimental setup is motivated.

Simple tapping tasks were used in previous research on sensory-motor synchronization (SMS). Participants were asked to follow a constant rhythmic stimulation sequence mostly by finger tapping (for review, see e.g., Repp, 2005). Tapping performance is typically measured as mean asynchrony—time difference between the finger tap and the rhythmic stimulus. The difference is negative if the taps precede the stimuli. By using such a tapping task with rhythmic stimuli, previous studies have often reported negative mean asynchronies, although participants typically reported the subjective feeling of synchrony (Repp, 2005; Repp and Su, 2013). For tapping with auditory stimuli in a regular rhythm it is assumed that synchrony is established at higher cognitive (“central”) levels, and the negative values are due to different processing times for the different sensory modalities, here: the auditory pacer stimulus and the tap (Aschersleben and Prinz, 1995; Aschersleben, 2002). However, this account still needs to be detailed as the observed asynchronies depend on pacer modalities and the duration of the pacers (Aschersleben, 2002). Interestingly, tapping with rhythmic visual stimuli often shows larger variance than auditory-motor synchronization (Repp and Penel, 2002; Repp, 2003; Pollok et al., 2009). In addition, the lower limit of successful synchronization is about 400 ms for visual stimuli, compared to 150–200 ms for auditory stimuli (Repp, 2003). These modality-dependent differences were first attributed to the lower temporal resolution in the visual system, but recent studies utilizing a moving visual cue, i.e., a bouncing ball or up-down movement of a finger, observed a comparable tapping performance as with auditory clicks and better than with visual flashes (Hove et al., 2010, 2013a,b). Musical training is known to reduce the mean asynchrony in auditory SMS (Franěk et al., 1991; Drake et al., 2000b; Krause et al., 2010). However, it is unclear whether it also affects visuo-motor synchronization.

Accurate synchronization between a conductor and musicians in an orchestra is a joint action, which requires integration of simultaneous self- and other-related behavior leading to a certain action-perception coupling in a musician’s brain. This coupling may serve at least three cognitive functions: the first is to generate predictions about the outcome of one’s own and others’ movements (Sebanz et al., 2005; Atmaca et al., 2008; Sebanz and Knoblich, 2009), the second is to form the representation of actions by others (Keller et al., 2007; Novembre et al., 2012; Loehr et al., 2013), and the third is to integrate the co-actor’s action with the self-generated action (Novembre et al., 2014). In addition, staying in synchrony with others—interpersonal synchrony—is also discussed as interest of individuals to show their affiliation to group (Pecenka and Keller, 2011; Cacioppo et al., 2014). Their results suggest that knowing what a partner will do by prediction of the partner’s action is a cue for synchronized action. Interestingly, several studies in sports have further reported that expertize improves the ability to perceive and understand the behavior of opponents (Abernethy, 1990; Singer et al., 1996; Helsen and Starkes, 1999; Savelsbergh et al., 2002; Shim et al., 2005). A review paper also showed that experienced athletes are better than an amateur at detecting perceptual cues for prediction of other’s actions (Mann et al., 2007). Based on this evidence, we hypothesize that orchestra musicians are superior to nonmusicians in synchronization especially when under the guidance of a conductor.

Neuroimaging studies have reported that subcortical and cortical areas whose functions range from basic timing processes to motor planning and action, such as the basal ganglia, the cerebellum, the thalamus, the motor cortex, and the supplementary motor area (SMA; Lewis and Miall, 2003; Rubia and Smith, 2004; Witt et al., 2008; Mendoza and Merchant, 2014; Merchant et al., 2015). Note, that studies on synchronous tapping of non-human primates show firstly that also monkeys can perform such tasks ideally under visual pace markers and secondly that their medial premotor areas host timer-like neurons measuring both, the time from the last marker as well as the expected time to the next marker. For a deeper discussion see the review by Merchant and Honing (2014). Although auditory and visual tapping tasks activate common brain areas such as the motor cortex, the SMA, and the cerebellum, the visual task recruits additional areas, including the ventral premotor cortex (vPMC), the insula, the putamen, and the inferior frontal gyrus (IFG; Jäncke et al., 2000; Jantzen et al., 2005; Pollok et al., 2009; Repp and Su, 2013). While musical experience increases the functional connectivity between the PMC and the thalamus in auditory-motor synchronization (Krause et al., 2010), it is unknown whether musical experience, especially the frequency of playing music under a conductor, affect the brain regions related to visuo-motor synchronization.

Current literature on the neural correlates of interpersonal synchrony report several brain regions being involved in successful synchronization. Neuroimaging studies have demonstrated that gesture recognition and imitation activates fronto-parietal areas, including the IFG and the inferior parietal lobe (IPL; Iacoboni et al., 1999; Hermsdörfer et al., 2001; Buccino et al., 2004; Chaminade et al., 2005; Mühlau et al., 2005; Pazzaglia et al., 2008; Villarreal et al., 2008; Green et al., 2009). These regions are known as a core of the mirror neuron network (Iacoboni and Dapretto, 2006; Cattaneo and Rizzolatti, 2009). The mirror neuron network is involved in both action observation and execution, leading to the concept that we interpret the actions of others by mimicking them mentally. A further region is the medial prefrontal cortex (mPFC), which is consistently activated when we think about other people’s mental states (Frith and Frith, 1999; Amodio and Frith, 2006). In particular, the anterior medial part of the superior frontal gyrus (SFG) is activated by mental simulation of a partner’s action (Decety et al., 1994, 1997; Grezes, 1998; Amodio and Frith, 2006). This region is also active during gestural communication and being in synchrony (Sebanz et al., 2007; Schippers et al., 2010; Fairhurst et al., 2013; Cacioppo et al., 2014). These results suggest that activity in the mPFC reflects successful mental simulation and more effective synchronized action.

Based on this evidence, we hypothesized that the effect of experience on predicting a partner’s action would be reflected by the activity in the mPFC, particularly the SFG, as a result of more precise mental simulation than their inexperienced counterparts. This would also be the case for synchronization between a conductor and orchestral musicians. To elucidate, we measured brain activity using functional magnetic resonance imaging (fMRI) while orchestral musicians and nonmusicians performed a synchronized tapping task under the guidance of a conductor’s gestures. Silent movies of conductor’s gestures were chosen as stimuli as we had planned to have the stimuli as realistic as possible for musicians. It was one of our concerns that musicians might show their expertize only when they followed a conductor’s gestures, but not during a simple tapping task with mechanical stimuli. Therefore we also designed a synchronized tapping task with a swinging metronome to investigate whether expertize effects in synchronized tapping are use-dependent or general improvement of sensitivity in timing processing. In addition, perturbation of rhythm was included in the task to evaluate how the brain areas associated with sensory-motor coordination respond to temporal modulation. We were interested in comparing differences between experts and novices using two groups of stimuli—the conductors as the stimulus taken from the field of expertize and the metronome as a somewhat related, though mechanical replacement.

## Materials and Methods

### Participants

Eleven participants who regularly played musical instruments in an orchestra (musicians: 6 males and 5 females) and 14 participants who have neither experience in playing music under a conductor or learn how to play a musical instrument (nonmusicians: 11 males and 3 females) participated in the experiment. All participants were right-handed, according to the Edinburgh Handedness Inventory (Oldfield, 1971) and had a mean age of 25 ± 3 years. They were paid for their participation and gave prior written informed consent. Procedures were conducted in accordance with the Declaration of Helsinki and the guidelines were approved by the Ethics Committee of the University of Leipzig. Musicians were members of an amateur orchestra and played music under a conductor regularly for 1–9 h per week (Mean ± SD: 4 ± 2) over the past 5 years, having at least 9 years of experience (M ± SD: 14 ± 4 years) in playing a musical instrument: violin, cello, contrabass, flute, trumpet, or trombone. Following the experiment, the musicians were asked how frequently they use a metronome during practice. Eight musicians used a digital metronome that only produced click sounds, but none of the musicians used an analog metronome with a swinging bar. None of the musicians practiced frequently with their metronome (M ± SD: 1.9 ± 0.9 on a 5-point scale; 1 = “*do not use at all*” and 5 = “*everyday use*”).

### Stimuli

**Figure 1:**
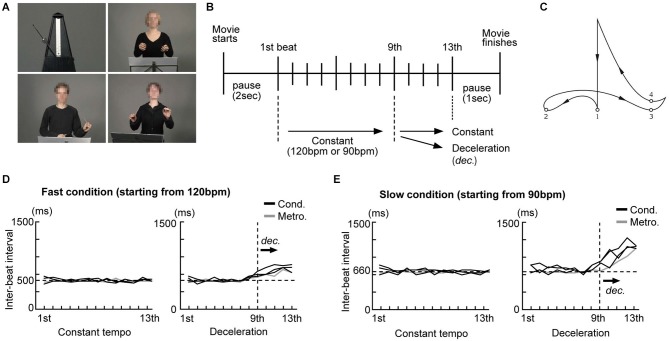
**Examples of the stimuli and the experimental design. (A)** Pictures taken from the silent movies of an analog metronome and three conductors were presented. The conductors’ faces were blurred to avoid brain activity related to facial expressions. **(B)** Schema of beat presentation. The inter-beat intervals (IBI) under all conditions were kept constant up to the 9th beat. Under the constant condition the IBI was not changed up to the last beat. Under the deceleration condition, the IBI was prolonged from the 9th beat onwards. **(C)** Conductors’ typical arm trajectory presenting each beat in a quadruple. The small numbered circles represent the points that, according to the literature, are used to indicate each beat. **(D)** The time course of the IBI of the metronome movements and conductors’ gestures under the fast condition (starting from 120 bpm) **(E)** The time course of the IBI of the metronome movements and the conductors’ gestures under the slow condition (starting from 90 bpm).

Conducting performances of three different conductors (one male and two females) and a swinging metronome (Figure 1A) were filmed without sounds using a digital video camera (Sony HDR-HC1E). The conductors were instructed to perform conducting gestures as they normally do. We selected 120 beats per minute (bpm) (500 ms inter-onset interval (IOI), fast condition) and 90 bpm (667 ms IOI, slow condition) as starting speeds as these are within the range of rates (400–800 ms IOI) known to yield reliable beat perception and are optimal for synchronized tapping (Drake et al., 2000a; McAuley et al., 2006). Each conductor was recorded performing two different conducting styles (constant tempo and deceleration: Figure 1B) at 120 and 90 bpm. For the constant tempo style, they were asked to maintain the speed until the last beat. For the deceleration style, they were asked to decelerate the speed during the last four beats either from 120 bpm to 90 bpm or from 90 bpm to 60 bpm, like a *ritardando* as they usually do in live performance. After some practice with a metronome, they conducted in each style without any external reference and filming took place. The movies were edited using Final Cut Pro (ver. 6, Apple Inc.) and the timing of each beat was calculated. A conductor’s gestures generally follow a certain pattern and each beat is normally presented when the arm reaches the lowest point of each arm movement (Farberman, 1997; Luck and Nte, 2008). Thus, we defined the lowest point of the arm movements as the representation of each beat and estimated the latencies of that as reference times for each beat representation (Figure 1C).

For the metronome stimuli, short movies of a swinging metronome (Figure 1A, upper left) were initially filmed at nine different speeds (from 120, 112.5, 105 … to 60 bpm, using steps of 7.5 bpm). Since we recorded the metronome without sound, we were free to define the representation of beats as those moments in time when the bar of the metronome was located at the extreme left and right. All movies started from either 120 or 90 bpm. For the deceleration style, movies were kept at a starting speed for the first nine beats and the speed was gradually decelerated from the 10th beat to the last (13th). The impression of metronome deceleration was created as follows: The movies of the metronome at different speeds were first split into fragments showing one beat and arranged sequentially from the starting speed down to the final speed. The final movie for the deceleration style starting at 120 bpm was created as follows: the first nine beats were presented at 120 bpm, the 10th beat at 112.5 bpm, the 11th beat at 105 bpm, the 12th beat at 97.5 bpm and the final beat at 90 bpm.

The timing of each beat presented under all conditions (from the 1st to the last) was calculated by the number of frames (40 ms/frame) from the beginning of the movie. Please note that the steepness of deceleration differed between the fast condition (starting from 120 bpm) and the slow condition: deceleration started from 120 bpm (500 ms IOI) to 90 bpm (667 ms IOI) under the fast condition, while it started from 90 bpm (667 ms IOI) to 60 bpm (1000 ms IOI) under the slow condition. That is, the deceleration under the slow condition was steeper than that under the fast condition. Figures 1D,E show the time course of the inter-beat interval (IBI) of the stimuli under the constant tempo and deceleration conditions. While the IBI under the constant tempo condition appears to be similar between the conductors and the metronome, the deceleration condition showed variability among the conductors. We did not match the IBI between the conductors as we considered this variability critical for our investigation into the effects of expertize when tapping under the guidance of a conductor.

### Procedure

During the fMRI scan, participants were required to synchronize the timing of tapping with their right index finger, with the timing of each beat presented by either the metronome or the conductors in the silent movie stimuli. The length of each stimulus was about 10 s in the fast condition and 12 s in the slow condition. Before entering the scanner, they trained to familiarize with the task. When training with the conductors’ gestures, they were instructed to tap when the arm of the conductor was at the lowest point of each arm movement. When training with the swinging metronome, they were instructed to tap when the bar of the metronome reached extreme left and right positions. All participants understood the instruction without any problems. We checked their tapping performance visually and told them whether their tapping action was correct or not. The overall length of training lasted for 15 min and all participants performed as instructed. During the scan, participants lay supine on an MRI scanner bed with their right index finger placed on a custom-made tapping pad. The movies were projected onto a back projection screen via a video projector (Panasonic PT-D7700E). The timing of taps was measured using a custom-made air pressure sensor, which was connected to the tapping pad in the scanner room. The length of plastic tube connecting the sensor and pad was about 10 m and caused a delay of 67 ms. The sensor consists of two moving bars with an air-pressure sensor in between. Taps on the upper bar lead to a hardly perceivable noise when the finger lands on the bar. Additionally, the participants put a noise attenuating headphone because the scanner noise is normally over 100 dB. Thus, the participants were unable to hear their own taps during the experiment. Tapping performance was corrected by subtracting the delay from the timing of taps. In addition, a white cross was presented on the gray background for 12 s as the baseline (null) condition and the participants were requested to remain still at the time of recording. During the recording session, all stimuli were presented with 15 repetitions in a random order with a mean interval of 3 s jittering from 2 s to 4 s. Scans were conducted using an event-related design. The design of the experiment was a four-way mixed design with a between-subject factor of Group (musicians, nonmusicians) and three within-subject factors of Stim (conductor, metronome), Style (constant tempo, deceleration), and Speed (fast: starting from 120 bpm, slow: starting from 90 bpm).

### Behavioral Data Analysis

Participants’ tapping performance was assessed using the temporal asynchrony, which is the subtraction of the time of taps from the time of corresponding beats presented by either the metronome or the conductors. A negative value represents a tap earlier than the beat. The analysis was focused on the temporal asynchrony during the last four beats (from 10th to 13th) of each sequence as the first nine beats in both style conditions were presented in the same way. The mean and the standard deviation (SD) of the temporal asynchrony were estimated for each participant under each condition and exported to R software (ver. 2.15.02). For ANOVA, we used an R package named “anovakun” produced by Dr. Ryuta Iseki. We conducted a four-way ANOVA with factors Group, Stim, Style, and Speed. *Post hoc* analyses were conducted using ANOVAs with pooled variances of the error terms from the original four-way model and Shaffer’s modified Bonferroni corrected *t*-tests (Shaffer, 1986).

#### fMRI Scan Acquisition

Data were acquired using a 3 Tesla Bruker Medspec 30/100 system with a standard birdcage head coil. Functional scans were collected as gradient echo, echo-planar imaging (EPI) with a blood oxygenation level dependent (BOLD) contrast (repetition time [TR] = 2000 ms, echo time [TE] = 30 ms, flip angle = 90°, field-of-view [FOV] = 19.2 cm^2^, matrix size = 64 × 64). Thirty slices which covered the cerebellum were acquired in one session with 3.0 mm thickness and an inter-slice gap of 1.0 mm, oriented perpendicular to the anterior-posterior commissure (AC-PC) line. Before the functional scan, a three-dimensional gradient-echo T1-weighted anatomical image of the whole brain (voxel size 1 mm^3^) was collected.

#### Data Pre-Processing

MRI data processing was conducted using Statistical Parametric Mapping (SPM8, Wellcome Trust Centre for Neuroimaging, University College, London, UK). Using the first slice as the reference, EPI images were corrected for slice timing and realigned spatially to the first image in the series using a 6-parameter affine transformation for motion correction (3 parameters for translation and rotation, respectively). The T1 image was co-registered to the mean EPI image. Then, the T1 image was normalized (using affine and smooth nonlinear transformations) to the brain template in Montreal Neurological Institute (MNI) space. The resulting normalization parameters were applied to the co-registered EPI images. Images were smoothed using an 8 mm^3^ full-width half-maximum Gaussian kernel.

#### Individual First-Level Analysis

First-level analysis was conducted using the general linear model. A statistical model for each participant was computed, applying a boxcar model, convolved with SPM’s canonical hemodynamic function (HRF). Motion correction parameters were entered into the model as covariates and the low frequency noise was removed with a 128 s high-pass filter. For each participant, statistical parametric maps of the *t*-statistic (SPM [T]) were generated by comparing each condition against the null condition. These *t*-maps were taken to second-level analysis.

#### Second Level Analysis

Contrast images of each participant were subjected to second-level random effect analyses. In order to visualize commonly activated brain areas during both constant conditions (starting from 120 bpm and 90 bpm), a conjunction analysis was performed. Each participant’s contrasts for both conditions against the null condition were used as the inputs to a second-level full factorial model. The obtained images were visualized with a threshold of cluster level FDR *p* < 0.05 and the cluster size of >100 voxels.

In order to identify regional brain activity modulated by the experience of playing music under the guidance of a conductor during synchronized tapping, we conducted separate three-way ANOVAs with factors Group, Style, and Speed for the conductor and metronome conditions. Follow-up comparisons were conducted using *t*-contrasts. To further investigate the interaction between the brain activity and musical experience, we conducted whole brain regression analyses using two kinds of musical experience as covariates: the number of years of playing musical instruments and the number of hours per week of playing under a conductor. A threshold was set for all statistical maps with a cluster level FDR *p* < 0.05. The surviving voxels were superimposed onto the MNI brain template. The voxel coordinates were converted to Talairach space using the GingerALE software (Laird et al., 2005). Anatomical labeling was provided by Talairach Client software (Lancaster et al., 2000).

## Results

### Behavioral Data

**Figure 2:**
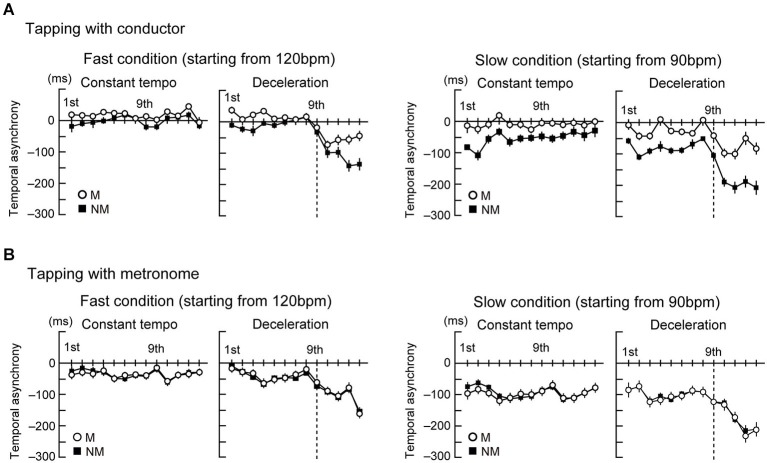
**The time course of the temporal asynchrony computed as difference in time between each corresponding pair of taps and beats**. Note that negative values represent taps being earlier in time than the corresponding beat. **(A)** The time course of the temporal asynchrony under the conductor condition.** (B)** The time course of the temporal asynchrony under the metronome condition. Error bars display standard error of the mean (*SEM*).

Figures 2A,B display the time course of the temporal asynchronies in the conductor and metronome conditions, respectively. The mean of the temporal asynchrony of the last four beats was analyzed by a four-way ANOVA with the factors Group, Stim, Style, and Speed. This analysis showed that the temporal asynchrony was smaller in tapping with the conductor than with the metronome (main effect of Stim: *F*_(1,23)_ = 20.8, *p* < 0.001, $\eta_{p}^{2}$ = 0.48). The asynchrony was also smaller while tapping in the constant tempo than in the deceleration (main effect of Style: *F*_(1,23)_ = 356.8, *p* < 0.001, $\eta_{p}^{2}$ = 0.94) and smaller in the fast condition than the slow condition (main effect of Speed: *F*_(1,23)_ = 178.7, *p* < 0.001, $\eta_{p}^{2}$ = 0.86). Two-way interactions were found between Group × Stim (*F*_(1,23)_ = 10.4, *p* = 0.004, $\eta_{p}^{2}$ = 0.31), Group × Style (*F*_(1,23)_ = 10.1, *p* = 0.004, $\eta_{p}^{2}$ = 0.31), Group × Speed (*F*_(1,23)_ = 5.9, *p* = 0.023, $\eta_{p}^{2}$ = 0.21), Stim × Style (*F*_(1,23)_ = 31.8, *p* < 0.001, $\eta_{p}^{2}$ = 0.58), and Style × Speed (*F*_(1,23)_ = 17.7, *p* < 0.001, $\eta_{p}^{2}$ = 0.45). In addition, there was a three-way interaction between Group, Stim, and Style (*F*_(1,23)_ = 24.1, *p* < 0.001, $\eta_{p}^{2}$ = 0.51), Group, Style, and Speed (*F*_(1,23)_ = 18.1, *p* < 0.001, $\eta_{p}^{2}$ = 0.44), and Stim, Style, and Speed (*F*_(1,23)_ = 7.3, *p* = 0.012, $\eta_{p}^{2}$ = 0.24), and a four-way interaction (*F*_(1,23)_ = 7.7, *p* = 0.011, $\eta_{p}^{2}$ = 0.25) was also found.

**Figure 3:**
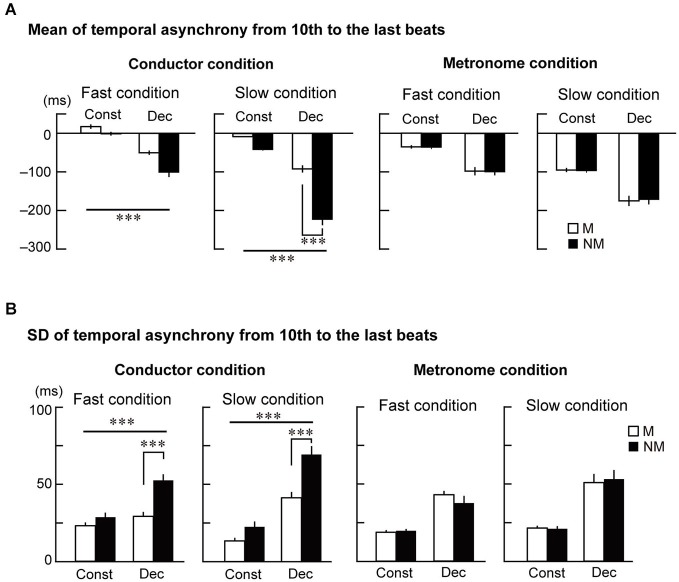
**The mean and standard deviation (SD) of the temporal asynchrony from the 10th to the last beat. (A)** The mean of each participant’s temporal asynchrony. **(B)** The mean of each participant’s SD of the temporal asynchrony. Error bars display standard errors of the mean (SEM). The triple asterisks represent *p* < 0.001. The abbreviations mean: M = musicians, NM = nonmusicians, Const = constant tempo condition, Dec = deceleration condition.

As follow-up analysis, we conducted two separate three-way ANOVAs with factors Group, Style, and Speed for the conductor and the metronome conditions using the error term of the first four-way ANOVA. In the conductor condition (Figure 3A left), this analysis showed that the temporal asynchrony in musicians was smaller than nonmusicians (main effect of Group: *F*_(1,23)_ = 8.1, *p* = 0.009, $\eta_{p}^{2}$ = 0.31). Also, the asynchrony was smaller while tapping in the constant tempo than in the deceleration (main effect of Style: *F*_(1,23)_ = 258.9, *p* < 0.001, $\eta_{p}^{2}$ = 0.91) and smaller in the fast condition than the slow condition (main effect of Speed: *F*_(1,23)_ = 75.7, *p* < 0.001, $\eta_{p}^{2}$ = 0.81). There were also two-way interactions of Group × Style (*F*_(1,23)_ = 21, *p* < 0.001, $\eta_{p}^{2}$ = 0.46), Group × Speed (*F*_(1,23)_ = 13, *p* = 0.001, $\eta_{p}^{2}$ = 0.42), and Style × Speed (*F*_(1,23)_ = 21, *p* < 0.001, $\eta_{p}^{2}$ = 0.39). Further follow-up ANOVAs with the factors Group and Style in the two speed conditions separately showed main effects of Group (*F*_(1,23)_ = 13.8, *p* = 0.001, $\eta_{p}^{2}$ = 0.19) and Style (*F*_(1,23)_ = 78.6, *p* < 0.001, $\eta_{p}^{2}$ = 0.82) in the fast condition. In the slow condition, there were main effects of Group (*F*_(1,23)_ = 8.2, *p* < 0.001, $\eta_{p}^{2}$ = 0.38) and Style (*F*_(1,23)_ = 192.9, *p* < 0.001, $\eta_{p}^{2}$ = 0.87), and an interaction between them (*F*_(1,23)_ = 24.2, *p* < 0.001, $\eta_{p}^{2}$ = 0.46). *Post hoc* *t*-tests revealed significant difference between musicians and nonmusicians in the deceleration condition (*t*_(23)_ = 4.7, *p* < 0.001, *η*^2^ = 0.69). These results in the conductor conditions indicate the musicians’ superiority in tapping with a conductor, compared to nonmusicians. In addition, tapping under slow speed (slow condition and deceleration) made it difficult to keep in synchrony. The Group × Style interaction in the slow condition indicated that nonmusicians felt more difficult to synchronize with the conductors than musicians.

In the metronome condition (Figure 3A right), however, the three-way ANOVA showed only main effects of Style (*F*_(1,23)_ = 112.9, *p* < 0.001, $\eta_{p}^{2}$ = 0.93) and Speed (*F*_(1,23)_ = 104.1, *p* < 0.001, $\eta_{p}^{2}$ = 0.88). There was neither a main effect of Group or any significant interactions. Hence, we did not observe significant differences between musicians and nonmusicians when tapping with the metronome. Instead, changes to the metronome’s Speed and Style changed the task’s difficulty, as evidenced by the differences in the temporal asynchrony.

Figure 3B displays the SD of the temporal asynchrony during the last four beats under the conductor and metronome conditions. The SDs were analyzed using the four-way ANOVA and showed that the variance of temporal asynchrony in musicians was smaller than nonmusicians (main effects of Group: *F*_(1,23)_ = 10.9, *p* = 0.003, $\eta_{p}^{2}$ = 0.32). The variance was also smaller while tapping in the constant tempo than in the deceleration (main effect of Style: *F*_(1,23)_ = 181.2, *p* < 0.001, $\eta_{p}^{2}$ = 0.89) and smaller in the fast condition than in the slow condition (main effect of Speed: *F*_(1,23)_ = 6.1, *p* = 0.021, $\eta_{p}^{2}$ = 0.21). Two-way interactions were observed between Group × Style (*F*_(1,23)_ = 4.6, *p* = 0.044, $\eta_{p}^{2}$ = 0.17) and Group × Stim (*F*_(1,23)_ = 19.3, *p* < 0.001, $\eta_{p}^{2}$ = 0.46). There was also a three-way interaction between Group × Stim × Style (*F*_(1,23)_ = 9.5, *p* = 0.005, $\eta_{p}^{2}$ = 0.29).

Similar to the analysis of the mean of the temporal asynchrony, the SDs were analyzed using three-way ANOVAs in the conductor and metronome conditions separately. In the conductor condition (Figure 3B left), main effects of Group (*F*_(1,23)_ = 30.2, *p* < 0.001, $\eta_{p}^{2}$ = 0.56) and Style (*F*_(1,23)_ = 198.7, *p* < 0.001, $\eta_{p}^{2}$ = 0.90) were found. An interaction between them was also significant (*F*_(1,23)_ = 24.0, *p* < 0.001, $\eta_{p}^{2}$ = 0.52). Although a main effect of Speed did not reach significant, an interaction between Style and Speed was found (*F*_(1,23)_ = 32.0, *p* < 0.001, $\eta_{p}^{2}$ = 0.59). Further follow-up ANOVAs with the factors Group and Style in the two speed conditions were conducted. In the fast condition, this analysis showed main effects of Group (*F*_(1,23)_ = 14.3, *p* = 0.001, $\eta_{p}^{2}$ = 0.38) and Style (*F*_(1,23)_ = 38.7, *p* < 0.001, $\eta_{p}^{2}$ = 0.63), and an interaction between them (*F*_(1,23)_ = 13.3, *p* = 0.001, $\eta_{p}^{2}$ = 0.37). *Post hoc* *t*-tests revealed significant difference between musicians and nonmusicians in the deceleration condition (*t*_(23)_ = 4.5, *p* < 0.001, $\eta_{p}^{2}$ = 0.69). In the slow condition, there were main effects of Group (*F*_(1,23)_ = 13.4, *p* = 0.001, $\eta_{p}^{2}$ = 0.45) and Style (*F*_(1,23)_ = 160.4, *p* < 0.001, $\eta_{p}^{2}$ = 0.87), and an interaction between them (*F*_(1,23)_ = 10.1, *p* = 0.004, $\eta_{p}^{2}$ = 0.30). *Post hoc* *t*-tests revealed significant difference between musicians and nonmusicians in the deceleration condition (*t*_(23)_ = 3.94, *p* < 0.001, *η*^2^ = 0.63). These results in the conductor conditions indicate that nonmusicians’ tapping was more variable than musicians, especially in the deceleration conditions. In the metronome condition (Figure 3B right), on the other hand, the three-way ANOVA only showed main effects of Style (*F*_(1,23)_ = 72.2, *p* < 0.001, $\eta_{p}^{2}$ = 0.76) and Speed (*F*_(1,23)_ = 11.5, *p* = 0.003, $\eta_{p}^{2}$ = 0.33). These results also indicate that there was no expertize effect in tapping with the metronome, and that the tempo and speed change of the metronome made synchronized tapping more variable in both musicians and nonmusicians.

**Figure 4:**
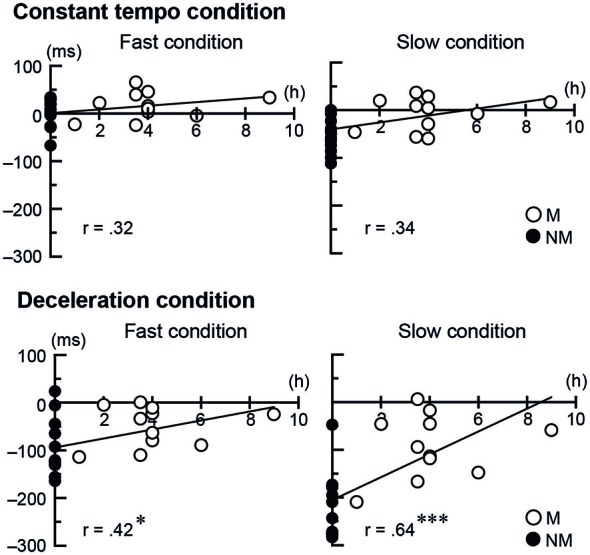
**The scatter plots of each participant’s temporal asynchrony under the conductor conditions and the frequency of playing music with a conductor per week**. The single and triple asterisks represent *p* < 0.05 and < 0.001, respectively. The abbreviations mean: M = musicians, NM = nonmusicians.

To further investigate the musicians’ expertize effect in the tapping performance, for both Stim conditions (conductor and metronome) we conducted separate correlation analysis between the temporal asynchronies and two kinds of musical experience: one being the number of years playing musical instruments and the other being the number of hours per week playing music under a conductor. The number of years playing musical instruments did not show correlation with the temporal asynchrony, neither for the conductors nor for the metronome. The number of hours per week playing music with a conductor, however, showed positive correlation in the deceleration conditions when tapping with the conductor (fast speed: *r* = 0.42, *t*_(23)_ = 2.23, *p* = 0.036, 95% CI [0.71 0.01]; slow speed: *r* = 0.64, *t*_(23)_ = 4.02, *p* < 0.001, 95% CI [0.83 0.31]). This indicates better synchronization with more frequent practice (Figure 4). The number of hours per week playing music with a conductor did not show significant correlation when tapping with the metronome.

To summarize the behavioral analysis, synchronized tapping was more challenging under the slow and deceleration conditions. Nevertheless, musicians showed higher accuracy of synchronization under the conductor than nonmusicians, which also correlates with the frequency of playing music with a conductor. In contrast, tapping with the metronome did not show any difference between musicians and nonmusicians.

### fMRI Data

**Figure 5:**
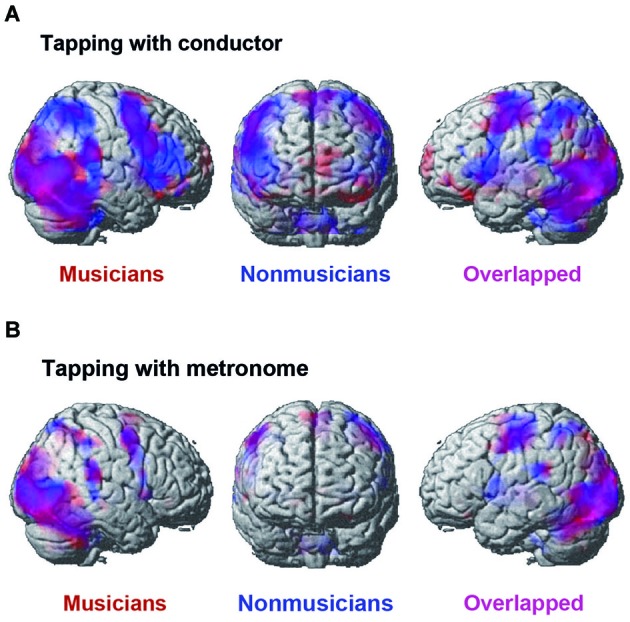
**The brain areas that were activated by the conjunction analysis of the constant tempo condition (120 bpm and 90 bpm). (A)** The activated areas under the conductor condition. **(B)** The activated areas under the metronome condition. A threshold was set at cluster level FDR of *p* < 0.05 and a cluster size of more than 100 voxels for all activated voxels. Activation in musicians (red) and nonmusicians (blue) are superimposed on the MNI template brain.

Figure 5 displays the activated areas in the constant tempo condition while the participants kept in synchrony either with the conductor or with the metronome. A number of brain areas were found active in the conductor condition: the middle occipital gyrus (MOG), the motor areas including the pre-/post central gyrus and the SMA, widely distributed fronto-parietal areas, including the IFG and the IPL, and the cerebellum. There was also activation in the subcortical areas, including the thalamus, the insula, and the basal ganglia, although these areas are not shown in Figure 4. On the contrary, the activated areas in the metronome conditions were relatively small, but included similar areas as those found in the conductor condition, namely; the occipital lobe, the pre-/post central gyrus, the cerebellum, and the subcortical areas, including the thalamus, insula, and the basal ganglia.

**Figure 6:**
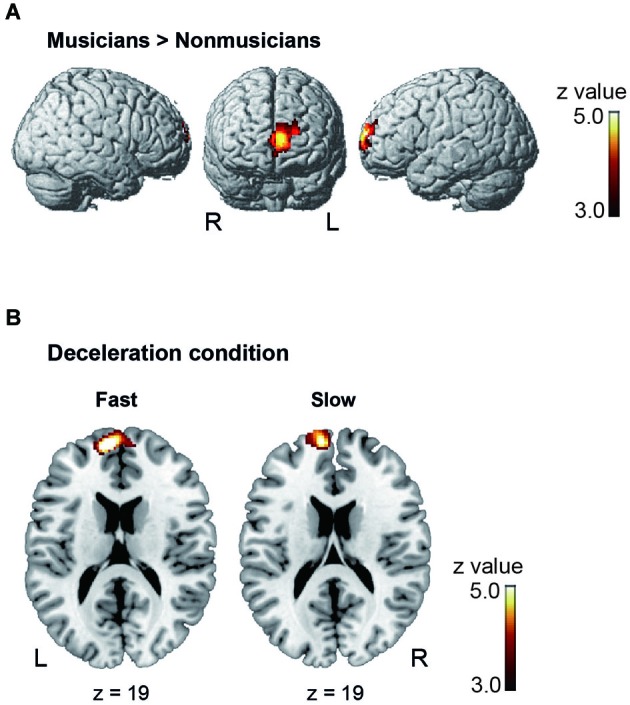
**The brain areas showed significant activity under the conductor condition (cluster level FDR *p* < 0.05). (A)** The brain areas showing significantly stronger activity in musicians than nonmusicians in the *post hoc* *t*-contrast between musicians and nonmusicians. **(B)** The brain areas significantly correlated with the number of hours playing music weekly under a conductor under the deceleration condition.

**Figure 7:**
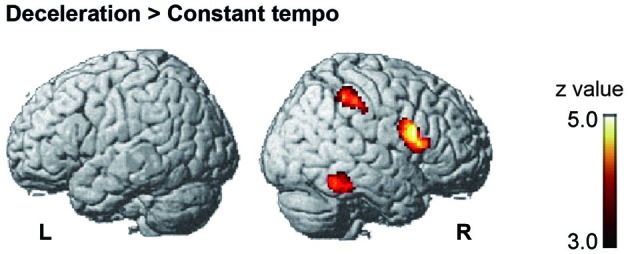
**The brain areas showed significant activity in the *post hoc t*-contrast between the constant tempo and deceleration conditions under the metronome condition (cluster level FDR corrected *p* < 0.05)**.

Under the conductor condition, the three-way ANOVA only revealed a main effect of Group. Thus, we created *t*-contrasts between musicians and nonmusicians to brain regions being more strongly activated in either group. The left SFG was identified with stronger activity for musicians than nonmusicians (Figure 6A). There was no brain region with stronger activity for nonmusicians than musicians. Under the constant tempo condition, planned whole brain regression analyses with two kinds of musical experience did not show any correlated brain areas. In the deceleration condition, however, the regression analysis with the number of hours per week playing music with a conductor showed positive correlation in the anterior part of SFG/MFG (Figure 6B). These results indicated that playing music more frequently under the guidance of a conductor leads to stronger SFG/MFG activity, at least under the condition in which the conductors decelerated the tempo. On the other hand, the three-way ANOVA in the metronome condition only showed a main effect of Style. The *t*-contrasts between the constant tempo and deceleration conditions showed stronger activity in the right IFG, IPL, and the fusiform gyrus (FG) for the deceleration condition than the constant tempo condition (Figure 7). The peak coordinates of the *t*-contrasts, shown in Figures 6A, 7, are listed in Table 1.

**Table 1:** **Peak coordinates of significantly activated areas (FDR corrected *p* < 0.05) in the *t*-contrast related to GROUP and STYLE**.

Brain area (Brodmann’s Area [BA])	Peak coordinates			Number of voxels	*z* value
	*x*	*y*	*z*
**Tapping with the conductor**
**Group: Musicians > Nonmusicians**
Left Superior Frontal Gyrus (BA10)	−4	66	22	591	4.15
**Tapping with the metronome**
**Style: Deceleration > Constant tempo**
Right fusiform gyrus (BA37)	48	−46	−18	582	4.76
Right inferior forntal gyrus (BA44)	54	18	12	524	4.68
Right inferior parietal lobe (BA40)	54	−36	44	443	4.00

## Discussion

The present study investigated visuo-motor synchronization in musicians and nonmusicians using movies of a conductor’s gestures and a swinging metronome. Behavioral performance showed that musicians’ tapping following a conductor’s gestures was synchronized more precisely than tapping with the metronome. The superiority of musicians’ tapping was observed in the conductor condition, especially when the conductors decelerated the tempo. Furthermore, fMRI results showed that the frequency of playing music with a conductor had a significant influence on the activity in the anterior part of the SFG/MFG, especially when the conductor changed the tempo during the tapping task. In contrast, when tapping with the metronome, neither behavioral performance nor brain activity showed significant differences between musicians and nonmusicians.

### Temporal Asynchrony and Effects of Experience Playing Music Under a Conductor

In the present study, using complex human and mechanical motions as visual stimuli, we observed the negative mean asynchrony as in the previous studies (Repp, 2005; Luck and Sloboda, 2008; Repp and Su, 2013). Additionally, musicians produced small positive asynchrony (less than 50 ms) while tapping with the conductor in the fast constant tempo (Figures 2A, 3A left). This may be an interesting observation for research on models of SMS, but we suggest that this positivity is still evidence of predictive tapping because the value of positivity is shorter than possible reaction time (about 100 ms). Tapping performance under the conductor condition meets our hypothesis that orchestra musicians are better to synchronize with a conductor than nonmusicians. The previous studies in sports suggested that better performance in experts is based on better prediction of opponents’ actions (Abernethy, 1990; Singer et al., 1996; Helsen and Starkes, 1999; Savelsbergh et al., 2002; Shim et al., 2005; Mann et al., 2007). The deceleration conditions in tapping under the conductor (Figure 2A) appear that the temporal asynchrony in musicians increased after the 10th beat and remained at this level, while the asynchrony in nonmusicians increased. Together with expertize effects in sports, this difference between musicians and nonmusicians may reflect musicians’ higher proficiency in predicting the conductor’s gestures than nonmusicians. Correlations between the temporal asynchrony and the frequency of playing music under the guidance of a conductor may support this interpretation.

Although the effect of musical experience in tapping under a conductor is comparable with the previous tapping studies (Franěk et al., 1991; Chen et al., 2008; Luck and Nte, 2008; Repp, 2010), the results under the metronome condition did not show any effect of musical experience. The difference between conductor and metronome conditions may be due to the lack of familiarity and experience in the case of the metronome. Generally, an analog metronome produces click sounds, which is normally used as the primary cue to synchronize action. Thus, synchronized action with the silent metronome as in the present study was completely new for both musicians and nonmusicians. The lack of an expertize effect for the metronome condition shows that musicians’ superiority in tapping with the conductor is not due to an improvement of visuo-motor coordination in general. Rather, the improvement was achieved in a task-specific manner and the experience of playing music with a conductor is a crucial factor for precise synchronization with the conductor’s gestures.

### Impact of Frequency of Playing Music Under a Conductor on Brain Activity

The present study found that tapping under the conductor activates the left SFG/MFG in musicians more strongly than in nonmusicians. This supports our hypothesis about the experience of playing music under a conductor and the activity in the mPFC. These regions are known as a part of the network for social interaction. In particular, several studies reported that these regions are activated when an observer mentally simulates a partner’s actions (Decety et al., 1994; Grafton et al., 1996; Grezes, 1998) and predicts the intention of a partner’s gestures (Grèzes et al., 2004; de Lange et al., 2008; Centelles et al., 2011; Liew et al., 2011; Spunt et al., 2011). In addition, being in synchrony activates these regions more than being out-of-synchrony in a tapping task (Fairhurst et al., 2013; Cacioppo et al., 2014). Similar processes should occur during tapping with the conductor. Better tapping performance and stronger activity in the SFG/MFG in musicians suggest that musicians had more precise mental simulation for tapping under the conductor condition than nonmusicians. This is represented by positive correlations between the activity in the SFG/MFG and the frequency of playing music under a conductor (Figure 5B). Interestingly, the mPFC, including the SFG/MFG, is also related to “mentalizing”, which is the ability to represent another person’s psychological perspective (Frith and Frith, 1999; Amodio and Frith, 2006). Frith and Frith (1999) suggested three components of the mentalizing function with corresponding brain areas: (1) the superior temporal sulcus (STS) for detection of the behavior of agents; (2) the inferior frontal areas for representations of goals; and (3) the anterior part of the SFG for simulation of another’s behavior with the representation of our own mental states. The STS is also involved in joint attention, such as following the gaze of a partner (Redcay et al., 2010). As the design of the present study does not allow specifying any relationship between synchronized action with a conductor and the mentalizing function, the distinct role in these areas remains an interesting question for future research.

### Brain Activity when Tapping with the Metronome and Effect of Tempo Change

Under the metronome condition, musicians and nonmusicians showed similar activity patterns. This mainly included the motor-related areas, visual areas, cerebellum, and the subcortical structures as shown in previous studies (Rubia and Smith, 2004; Wiener et al., 2010; Merchant et al., 2013a, 2015). Interestingly, non-human primates also showed spike activity in the corresponding areas of the SMA, the putamen, the premotor cortex while rhythmic tapping with a sequence of auditory/visual stimuli, possibly suggesting similar neural networks for synchronized action between species (Merchant et al., 2011, 2013b; Bartolo et al., 2014; Crowe et al., 2014; Merchant and Honing, 2014). In addition, the activity in the FG, the precentral gyrus, and the IPL increased with the tempo change. With regard to time management, two distinct systems have been suggested: automatic and cognitively controlled timing systems (Lewis and Miall, 2003). The automatic timing system involves brain regions within the motor network, including the motor cortex, SMA, and cerebellum. That being said, the cognitive controlled timing system involves brain regions that contribute to cognitive abilities, such as working memory or attention, within the prefrontal and parietal cortices. The deceleration conditions in the present study requires many more cognitive resources to follow the beats than the constant tempo condition, thus the observed difference between the deceleration and constant tempo conditions may reflect the contribution of the cognitive timing system.

Although behavioral performance showed an effect of deceleration under both conductor and metronome conditions, brain activity did not show corresponding changes under the conductor condition. As far as we are aware, no study has addressed which regions of the brain are related to the tempo change in human sequential action. Therefore, we are only able to speculate as to why the aforementioned results were obtained in the present study. There are several differences between the deceleration in the metronome and the conductor. One possible interpretation might be related to the difference in the familiarity with the tempo change between a conductor and a metronome. Before the experiment, no one had ever seen the deceleration by the metronome. Thus, the unique experience in the present study might strongly stimulate the brain regions related to the cognitive processing of temporal information. In addition, deceleration only occurred during the last 2 s of the movie stimuli. Considering the delay of the BOLD change after stimulation in general, our fMRI measurement might only detect the initial rise of the BOLD change by the deceleration. Although the effect of deceleration did not reach significant level under the conductor condition, a small spot was found in the right IFG with a relaxed threshold (uncorrected *p* < 0.005). This might indicate the initial rise in activity caused by deceleration under the conductor condition.

## Conclusion

The present study demonstrated that the frequency of playing music under the guidance of a conductor has an impact on visuo-motor synchronization following a conductor’s gestures. The results indicated better tapping performance while tapping under the conductor, which corresponded with the wide distribution of the brain activity, including the fronto-parietal areas. The fMRI results also indicated that the anterior part of the left SFG specifically was more engaged in musicians than nonmusicians while tapping under a conductor. One possible interpretation is that musicians predicted the timing of the beats by mental simulation from the conductor’s gestures. In contrast, tapping with the metronome showed effects relating to the temporal modulation in both musicians and nonmusicians. This might be comparable with the theory of the cognitively controlled timing system. These results suggest that frequent practice in playing music under a conductor improves orchestra musicians’ ability to mentally simulate a conductor’s gestures, leading to superior performance in synchronized tapping and stronger activity in the SFG than nonmusicians.

## Conflict of Interest Statement

The authors declare that the research was conducted in the absence of any commercial or financial relationships that could be construed as a potential conflict of interest.
